# Detection of native interferon-γ in nyala (*Tragelaphus angasii*): Towards diagnosing tuberculosis

**DOI:** 10.4102/ojvr.v86i1.1796

**Published:** 2019-11-06

**Authors:** Lezaan Roux, Alicia J. McCall, Anita L. Michel

**Affiliations:** 1 Department of Veterinary Tropical Diseases, Bovine Tuberculosis and Brucellosis Research Programme, Faculty of Veterinary Science, University of Pretoria, Onderstepoort, South Africa; 2 Department of Agriculture and Rural Development, Veterinary Services, KwaZulu-Natal Province, Hluhluwe, South Africa

**Keywords:** interferon gamma, *Mycobacterium bovis*, nyala, *Tragelaphus angasii*, tuberculosis

## Abstract

*Mycobacterium bovis* is the main cause of tuberculosis in wildlife. In South Africa, African buffaloes (*Syncerus caffer*) are a wildlife maintenance host while a number of other species are considered spillover hosts. Nyala (*Tragelaphus angasii*), a large antelope species from Southern Africa, is frequently traded and can be infected with *M. bovis*. Interferon gamma (IFN-γ) release assays that detect cell-mediated immune (CMI) responses to *M. bovis* infection have shown promise in elephants, rhinoceroses and buffaloes. The BOVIGAM^®^ assay is a commercial IFN-γ release assay designed to detect tuberculosis in cattle and has been validated in buffaloes. We tested the suitability of the BOVIGAM^®^ assay to detect native IFN-γ release in nyala. Blood samples collected from 17 nyalas were stimulated with different mitogens and IFN-γ release measured. We found that incubating whole blood with phorbol 12-myristate 13-acetate and calcium ionophore (PMA/CaI) resulted in the highest levels of IFN-y release. Samples stimulated with tuberculin purified protein derivatives of *M. bovis* (PPDb) and *M. avium* (PPDa) did not show significant IFN-γ production. An intradermal tuberculin test (IDT) and culture of tissues from 15 of the 17 culled nyala were also performed, which supported the findings of the BOVIGAM^®^ assay, suggesting the potential value of this assay for the diagnosis of tuberculosis in nyala.

## Introduction

Bovine tuberculosis (BTB), caused by *Mycobacterium bovis (M. bovis)*, has been diagnosed in numerous wildlife species. Some of these wildlife species act as maintenance hosts, including African buffalo (*Syncerus caffer*) and greater kudu (*Tragelaphus strepsiceros*), while others are considered potential maintenance hosts such as lion (*Panthera leo*) and warthog (*Phacochoerus africanus*), or spillover hosts including chacma baboon (*Papio ursinus*), leopard (*Panthera pardus*), impala (*Aepyceros melampus*) and other bovidae (Michel et al. 2015). Nyala is a bovid native to Southern Africa that is regularly sold and translocated in large numbers across the country. Currently, there is little knowledge on their role in the epidemiology of BTB, and to the best of our knowledge, there is currently only one report on the occurrence and diagnosis of bovine tuberculosis in nyala in South Africa (Hlokwe, Van Helden & Michel 2014).

Methods currently used to diagnose tuberculosis in wildlife include bacterial cultures and for some species cell-mediated immune (CMI) response assays such as the intradermal tuberculin test (IDT) and interferon gamma (IFN-γ) assays (Maas, Michel & Rutten 2013). Bacterial culture is the gold standard in the diagnosis of tuberculosis and is most successfully performed on post-mortem tissue samples; but it can take up to 8 weeks to make an initial analysis and requires additional tests, such as polymerase chain reaction (PCR), to identify the pathogen present. The IDT has been used in several wildlife species but requires the capture and immobilisation of animals twice over the period of 72 h and has not yet been validated for nyala (Cousins & Florisson 2005). The IFN-γ assay detects IFN-γ released from T-cells of an *M. bovis* infected host. The advantages of the IFN-γ assay are that only whole blood, not tissue, is needed for the analysis; the analysis can be carried out within days; and the animal is captured only once for blood collection. However, the IFN-γ assay is species or at least family specific and will have to be designed and/or optimised for wildlife species. Mitogens known to cause optimal IFN-γ release independent of the host’s infection status are used as positive controls in the IFN-γ assay, but the mitogen’s efficacy might also be species specific (Angkawanish et al. 2013; Maas et al. 2012; Morar et al. 2013; Schiller et al. 2009).

BOVIGAM^®^ is a commercial IGRA kit used to analyse IFN-γ release in cattle but has been successfully validated for buffalo plasma (Michel et al. 2011). We tested whole blood collected from nyala and stimulated with a variety of mitogens and bovine and avian tuberculin in order to identify the conditions for native IFN-γ release in this species. Culture and PCR results were used to verify the CMI responses.

## Materials and methods

### Animals and sample collection

Tissue and blood samples were collected from 15 and 17 nyalas, respectively, in the Phinda Private Game Reserve in northern KwaZulu-Natal for IFN-γ assay and skin test before being culled for population management purposes. Blood, 2 mL × 9 mL, was collected in heparin vacutainer tubes and stimulated within 8 hours. Upon completion of all testing procedures, the nyalas were humanely euthanised and tissue samples collected from lungs, head lymph nodes, thoracic lymph nodes and kept frozen at −20 °C.

### Interferon gamma assay

Aliquots of 1 mL blood were incubated, respectively, with Roswell Park Memorial Institute (RPMI) 1640 Medium cell culture media (negative control), purified protein derivative of bovine tuberculin (PPDb, 24 *µ*/ml), purified protein derivative of avian tuberculin (PPDa, 48 *µ*g/mL) and mitogens: pokeweed mitogen (PWM, 5 *µ*g/mL and 20 *µ*g/mL); phorbol 12-myristate 13-acetate and calcium ionophore (PMA/CaI, 100 ng/mL and 2 *µ*g/mL); concanavalin A (ConA, 10 *µ*g/mL); phytohemagglutinin (PHA, 10 *µ*g/mL) and at 37 °C for 24 h. The supernatants were harvested and stored at –20 °C. The IFN-γ assay was carried out according to the manufacturer’s instructions (BOVIGAM^®^ 1G, AsureQuality Australia Pty Ltd). Optical density (OD) values (OD at 450 nm – 650 nm) values of mitogen and antigen-stimulated samples were blanked by subtracting the values of the animals’ specific negative controls.

### Intradermal tuberculin test

Two sites on opposite sides of the neck were shaved and skin fold thickness measured with calipers followed by injection of 0.1 mL of PPDb and PPDa, respectively. The skin reactions were read after 72 h by measuring the increase in skin fold thickness and clinically evaluated.

### Bacterial culture and polymerase chain reaction

Tissue handling and culture were performed as described before (Michel et al. 2017). Acid fast bacterial colonies were subjected to a multiplex PCR method previously described by Kim et al. (2013). Polymerase chain reaction products were run on a 2% agarose gel at 100 V for minimum of 1 h. The DNA fragments were viewed using ChemiDoc™ XRS system (Bio-Rad Laboratories, South Africa).

### Ethical considerations

Ethical clearance to conduct this study was obtained from the Animal Ethics Committee (AEC) of the University of Pretoria on 22 August 2016 (ethical clearance number: V078-16).

## Results

**FIGURE 1:**
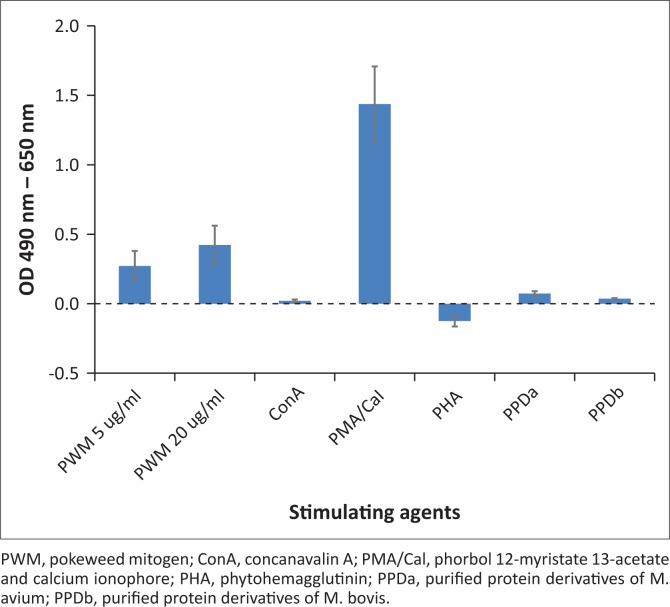
Optical densities of plasmas from nyala whole blood samples stimulated with different mitogens and antigens. Average blanked optical density values (490 nm – 690 nm) of mitogen or antigen-stimulated samples. Values were corrected by subtracting the antigen or mitogen values with the negative sample values of respective animals. Error bars represent standard error of the mean.

Whole blood samples (*n* = 17) were incubated, respectively, with PMA/CaI, both concentrations of PWM, ConA, PPDb and PPDa. Only two samples were incubated with PHA. PMA/CaI stimulation yielded the highest release of IFN-γ with blanked OD values ranging from −0.255 to 3.573 (Figure 1). Blanked OD ranges for samples incubated with 5 *µ*g/mL PWM, 20 *µ*g/mL PWM, ConA and PHA were −0.097 to 1.83, −0.04 to 2.112, −0.282 to 0.068 and −0.239 to −0.011, respectively. Negligible amounts of IFN-γ were detected in the antigen-stimulated samples, with OD values ranging from −0.259 to 0.016 for PPDb and −0.241 to 0.195 for PPDa.

The skin test was evaluated in 14 adult and one subadult nyala. The skin fold thickness at the bovine injection site remained unchanged in 11 animals and increased by between 0.2 mm and 0.5 mm in four animals. The skin fold thickness at the avian injection site remained unchanged in 10 animals and increased by between −0.3 mm and 0.4 mm in five animals. All animals showing a reaction at the bovine injection site also showed a reaction at the avian injection site. Subtraction of the avian increase from the bovine increase resulted in net increases of between −0.3 mm and 0.4 mm. With the exception of two animals which showed small hard swellings at the bovine injection site (net differential increases of 0.1 mm and 0.4 mm, respectively) none of the animals showed clinical signs at the injection sites. All skin test results were considered negative for bovine tuberculosis.

Bacterial culture of tissues from nyala 3, 5 and 17 showed growth on the Löwenstein-Jensen media after 10 weeks. The resulting bacterial colonies were all identified as non-tuberculous mycobacteria (Table 1).

**TABLE 1:** Results of bacterial culture (and polymerase chain reaction) of tissue samples from nyala.

Animal ID	Tissue type	Culture isolate (Yes/No)	PCR results
Nyala 1	Pooled body, head, mesenteric, hepatic, lymph nodes	N	-
Nyala 2 & 15	Pooled body, head, mesenteric, hepatic, thoracic lymph nodes, retropharyngeal lesion	N	-
Nyala 3	Pooled body, head, mesenteric, hepatic, thoracic lymph nodes, left bronchial lesion	Y	*Mycobacterium* species
Nyala 5	Pooled body, head, mesenteric, hepatic, thoracic lymph nodes	Y	*Mycobacterium* species
Nyala 6	Pooled body, head, mesenteric, hepatic, thoracic lymph nodes, thoracic lymph node lesion	N	-
Nyala 7 & 9–11	Pooled body, head, mesenteric, hepatic, thoracic lymph nodes	N	-
Nyala 12	Pooled body, head, thoracic lymph nodes	N	-
Nyala 13–14 & 16	Pooled body, head, mesenteric, hepatic, thoracic lymph nodes	N	-
Nyala 17	Pooled body, pooled head, mesenteric, hepatic, pooled thorax, lesion pool	Y	*Mycobacterium* species

PCR, polymerase chain reaction.

## Discussion

The increasing spread of *M. bovis* infection in wildlife in South Africa causes concern to game owners, conservation bodies and state veterinary services. The successful control relies on validated diagnostic assays for ante mortem testing of host species at risk of transmitting *M. bovis*.

CMI assays showed promise in detecting *M. bovis* infection in cattle and buffalo and were considered for potential use in nyala. This study aimed at determining which mitogen will result in sufficient T-cell stimulation and, therefore, optimal IFN-γ production. Not only did the PMA/CaI incubation result in the highest release of IFN-γ, but also the high OD values achieved showed that the BOVIGAM^®^ kit can be successfully used with nyala samples. This was even more evident in the antigen-stimulated samples that showed significantly and consistently lower OD values compared to the positive control. These results and conclusions indicated absence of exposure to *M. bovis* and were supported by the findings of the post-mortem examination, the IDT, bacterial culture and PCR results. The findings further demonstrate that the presence of non-tuberculous mycobacteria in three nyala did not cause any cross-reactions potentially resulting in false positive test results upon stimulation with PPDb in the BOVIGAM^®^ assay. In conclusion, the findings of this study highlight the potential value of the commercial BOVIGAM^®^ assay in the diagnosis of *M. bovis* infection in nyala and justify a validation study in this species.
